# Early Adverse Experiences and the Likelihood of Substance Use Disorders and Non-Fatal Overdose in Clinical and Community Settings: A Systematic Review and Meta-Analysis

**DOI:** 10.3390/bs16040589

**Published:** 2026-04-15

**Authors:** Olga Mariela Mogollón-Canal, Diana Janeth Villamizar-Carrillo, Sandra Licette Padilla-Sarmiento, Sandra-Milena Carrillo-Sierra, Anyela Patricia Villamizar-Carrillo, Javier Fernández-Montalvo, Omar Rozo-Pérez, Daniel Cepeda-Pineda, Diego Rivera-Porras

**Affiliations:** 1 Universidad de Pamplona, Facultad de Psicología, Grupo de Investigación Psicología y Sociedad, Pamplona 543050, Norte de Santander, Colombia; olgamariela@unipamplona.edu.co (O.M.M.-C.); jacarrillo71@unipamplona.edu.co (D.J.V.-C.); spadilla@unipamplona.edu.co (S.L.P.-S.); sandra.carrillo3@unipamplona.edu.co (S.-M.C.-S.); 2 Universidad de Pamplona, Facultad de Artes y Humanidades, Pamplona 543050, Norte de Santander, Colombia; anyela.villamizar@unipamplona.edu.co; 3 Universidad Pública de Navarra, Facultad de Ciencias de la Salud, 31001 Navarra, Spain; fernandez.montalvo@unavarra.es; 4 Universidad Simón Bolívar, Facultad de Ciencias Jurídicas y Sociales, Centro de Investigación en Estudios Fronterizos, Cúcuta 540001, Norte de Santander, Colombia; 5 Universidad de la Sabana, Facultad de Ciencias Del Comportamiento, Chía 250001, Cundinamarca, Colombia; danielcepi@unisabana.edu.co; 6 Universidad de la Costa, Departamento de Productividad e Innovación, Barranquilla 080001, Atlántico, Colombia

**Keywords:** adverse childhood experiences, childhood trauma, substance-related disorders, substance use disorders, non-fatal overdose, dual diagnosis, psychiatric treatment, meta-analysis

## Abstract

Early adverse experiences may increase the likelihood of substance use disorders/addictions (SUD) and overdose, but estimates vary across settings. This review synthesised observational evidence on early adverse experiences and disorder-level SUD outcomes and/or non-fatal overdose. PubMed, Scopus, Web of Science, ScienceDirect, SpringerLink, and Taylor & Francis were searched up until 1 December 2025. Eligible observational studies sampled psychiatric treatment or community populations, measured early adverse experiences, and reported associations with disorder-level SUD/addiction or non-fatal overdose. Risk of bias was assessed using Joanna Briggs Institute (JBI) tools. A random-effects meta-analysis pooled commensurate overdose estimates; the remaining evidence was synthesised narratively. Nine studies were included (n = 67,228 participants; one cohort did not report an analytic sample size). SUD associations were consistently positive (adjusted odds ratio (OR) range 1.71–4.30) but were not pooled due to heterogeneity in exposure and outcome definitions. Three studies (721 participants) contributed to the overdose meta-analysis; a higher adverse childhood experience (ACE) score was associated with higher odds of non-fatal overdose (pooled OR 1.16 per +1 ACE, 95% confidence interval (CI) 1.06–1.28; I^2^ = 28.4%). A Hartung–Knapp sensitivity analysis yielded OR 1.16 (0.95–1.41). Risk of bias was mostly moderate, commonly driven by self-reported outcomes and residual confounding. Early adverse experiences were associated with higher odds of SUD and non-fatal overdose across settings. Limitations include observational designs, variable measurement, and sparse poolable overdose evidence; future studies should distinguish fatal from non-fatal overdose and improve outcome harmonisation.

## 1. Introduction

Early adverse experiences—often operationalised as adverse childhood experiences (ACEs) that occur before age 18, including abuse, neglect, and household dysfunction—have become a central lens for understanding long-term vulnerability to health-harming behaviours. The original ACE study demonstrated graded associations between cumulative adversity and a wide range of adult outcomes, establishing a framework in which exposure intensity, rather than any single event, carries clinical significance (Felitti et al., 1998).

Across one’s lifespan, the link between early adversity and substance-related outcomes has been repeatedly observed, with cumulative exposure showing particularly consistent associations. A large meta-analysis pooled studies on multiple ACEs reported substantially elevated odds for problematic drug use among those with higher ACE counts, supporting the idea that adversity clustering is more informative than isolated exposures (Hughes et al., 2017). Complementary synthesis work has also documented markedly higher ACE burdens among people with substance abuse or addiction compared with general-population estimates, highlighting the relevance of ACE profiling when characterising clinical risk (Madigan et al., 2023).

At the population level, substance use and overdose continue to contribute substantially to morbidity and mortality. The World Health Organization (WHO) estimates that 296 million people used drugs at least once in 2021 and that 39.5 million people were living with drug use disorders; around 60 million people used opioids (World Health Organization, 2025). In 2019, approximately 600,000 deaths were attributable to drug use, and close to 80% of these deaths were opioid-related, with about 25% of opioid-related deaths caused by opioid overdose (World Health Organization, 2025). Across Organisation for Economic Co-operation and Development (OECD) countries, around 9% of people aged 15–64 reported illicit drug use in the last 12 months (2023 or nearest year), and opioid use disorders were responsible for nearly 74,000 deaths in 2022 (OECD, 2025).

Overdose outcomes are heterogeneous and often measured differently across studies. Opioid overdoses that do not lead to death are several times more common than fatal overdoses (World Health Organization, 2025). In addition, a history of non-fatal overdose is itself clinically meaningful: among people who inject drugs (PWID), prior non-fatal overdose has been associated with a higher subsequent risk of fatal overdose (Caudarella et al., 2016).

Mechanistically, early adverse experiences may influence later substance use and overdose through multiple pathways that potentially interact, including altered stress responsivity and emotion regulation, a higher prevalence of mood and anxiety disorders, and greater exposure to high-risk social environments. Meta-analytic evidence indicates substantial comorbidity between mood disorders and substance-related disorders (Saha et al., 2022), and a systematic review has reported associations between mental disorders and opioid overdose (van Draanen et al., 2022).

Large-scale observational evidence also supports a dose–response relationship between cumulative adversity and later psychopathology. In a Swedish twin cohort study, each additional adverse childhood experience was associated with increased odds of clinically confirmed adult psychiatric disorders (odds ratio (OR) per additional ACE 1.52, 95% confidence interval (CI) 1.48–1.57), and the association remained—attenuated but present—in discordant twin analyses (Daníelsdóttir et al., 2024). However, ACE measurement varies across studies (e.g., ACE checklists vs. trauma histories; prospective vs. retrospective ascertainment), which can complicate synthesis and may contribute to heterogeneity and risk of bias.

Parallel to the growing interest in using ACE histories as risk indicators, there is ongoing debate about the evidentiary basis for ACE screening as a stand-alone programme and about the need for adequate trauma-informed responses to screening results (Racine et al., 2020; Gentry & Paterson, 2022). This context underscores the importance of clarifying what can—and cannot—be concluded from existing evidence about ACEs in relation to substance-related outcomes and overdose.

Recent reviews have focused on opioid-related behaviours and opioid use disorder (OUD) specifically (Regmi et al., 2024; Deol et al., 2023; Meyer et al., 2023), yet overdose outcomes (particularly non-fatal overdose) and comparisons across clinical treatment settings versus community samples remain less consistently synthesised. Empirical work spans psychiatric treatment cohorts and safety-net or addiction-care settings (e.g., Gao et al., 2010; Bryant et al., 2020; Asheh et al., 2023) as well as community cohorts of people who inject drugs (Lake et al., 2015; Stein et al., 2017), but effect estimates remain difficult to compare when adversity constructs and outcome definitions are not commensurate.

Beyond heterogeneity in exposure and outcome definitions, setting-specific selection processes can shape observed associations. Clinical samples may over-represent individuals with a higher adversity burden and comorbid psychiatric symptoms and may rely on structured diagnostic assessments, whereas community samples may rely more on self-report and may capture earlier stages of substance involvement. Evidence from severe mental illness and dual-diagnosis contexts suggests that childhood trauma history can intersect with attachment and symptom severity when characterising substance-related risk (Campos et al., 2020), while population-based analyses continue to show elevated substance-related risk (SUD) odds among those with high ACE counts (Broekhof et al., 2023).

Clinical interpretation becomes more complex when substance use disorders (SUDs) are embedded within broader psychiatric presentations. Comorbidity between mood disorders and substance-related disorders is common and clinically consequential, with systematic evidence showing substantial overlap that can influence symptom course, service use, and outcome ascertainment (Saha et al., 2022). In such contexts, early adversity may intersect with psychiatric symptoms, social instability, and care pathways, producing heterogeneous risk profiles that are not well captured by evidence drawn solely from general community samples.

Overdose adds a second outcome domain that is clinically urgent yet methodologically distinct from disorder-level diagnoses. Opioid dependence and overdose mortality continue to impose considerable population-level harm, and global analyses have documented substantial opioid-related morbidity and mortality, while also noting variation by region and time period (Degenhardt et al., 2019). Evidence syntheses focused on opioids suggest that ACE exposure is associated with later opioid use-related behaviours, including opioid dependence and lifetime opioid overdose, with several studies describing graded risk as ACE counts rise (Regmi et al., 2024).

Despite this growing literature, two practical gaps remain for the decision context addressed here. Existing systematic reviews/meta-analyses have synthesised ACE exposure in relation to problematic substance use and opioid use-related behaviours, yet estimates are difficult to interpret for clinically defined SUD/addiction outcomes and overdose endpoints across psychiatric-treatment and community settings because endpoints and ascertainment methods are frequently combined (Hughes et al., 2017; Madigan et al., 2023; Regmi et al., 2024). First, many reviews focus on substance use behaviours rather than clinically defined SUD/addiction outcomes, limiting comparability with psychiatric services and epidemiological case definitions. Second, overdose outcomes are often pooled with other opioid behaviours or treated as a secondary endpoint without clear separation of fatal versus non-fatal events, without consistent reporting of ascertainment (self-report, clinical record, registry linkage), and without explicit comparison across psychiatric treatment populations and community-dwelling samples. These issues matter for evidence synthesis because exposure definition (ACE inventory vs. broader childhood trauma constructs), outcome definition (diagnosis vs. symptom threshold vs. event history), and confounding control can materially shift effect estimates in observational research. In this review, overdose outcomes primarily reflect non-fatal overdose history in living participants (often self-reported), rather than fatal overdose outcomes.

This systematic review addressed the following Population–Exposure–Outcome (PEO) question: In psychiatric patients receiving psychological, psychiatric, or integrated treatment, or in community-dwelling individuals (P), is exposure to early adverse experiences (E) associated with a higher likelihood of developing substance use disorders/addictions (primary outcome) or experiencing a non-fatal overdose episode (secondary outcome) (O)?

## 2. Materials and Methods

### 2.1. Design, Protocol, and Reporting Standards

This systematic review was planned as a synthesis of observational evidence on early adverse experiences and later substance-related outcomes. Reporting follows Preferred Reporting Items for Systematic Reviews and Meta-Analyses (PRISMA) (Page et al., 2021) and incorporates the literature search reporting elements specified in PRISMA-Search (PRISMA-S) (Rethlefsen et al., 2021). Given the observational nature of the evidence base, Meta-Analysis of Observational Studies in Epidemiology (MOOSE) principles were also used to structure the presentation of exposure–outcome associations and analytic decisions (Stroup et al., 2000).

Work began in September 2025. Study selection and metadata extraction were managed in Microsoft Excel. The review team comprised six investigators, organised into two independent groups of three; uncertainties arising at screening or extraction were resolved through joint discussion until consensus.

International Prospective Register of Systematic Reiews (PROSPERO) protocol registration: Rivera Porras DA, Mogollón Canal OM, Padilla Sarmiento SL, Villamizar Carrillo DJ, Villamizar Carrillo AP, Fernández Montalvo J, et al. Early adversities and mechanisms determining substance use trajectories and overdose risk: a systematic review. PROSPERO 2025 CRD420251250427. Available from https://www.crd.york.ac.uk/PROSPERO/view/CRD420251250427 (accessed on 18 December 2025). Protocol amendments: none reported (PROSPERO, 2025).

### 2.2. Review Question and Operational Structure (PEO)

The review question was framed using the Population–Exposure–Outcome (PEO) structure, appropriate for observational aetiology/risk questions (Hosseini et al., 2024).

Table 1 specifies the operational structure of the review question using the PEO framework, including the primary and secondary outcomes that governed eligibility and extraction decisions.

### 2.3. Eligibility Criteria

Eligibility criteria were set a priori to maintain alignment with the PEO question and to support quantitative synthesis when feasible. For synthesis, studies were grouped by outcome domain (SUD/addiction vs. non-fatal overdose); overdose studies were eligible for quantitative pooling only when they reported commensurate adjusted effect estimates per 1-point increase in ACE score.

Two clarifications were applied to prevent internal inconsistencies between eligibility rules and the included evidence base: (1) Age restriction (minors). Studies were excluded when the sampled population was restricted to individuals aged <18 years and the eligible outcomes (SUD/addiction and/or overdose) were measured within childhood/adolescence. Cohorts recruited during adolescence were eligible when early adversity was measured as the exposure and eligible outcomes were ascertained in adulthood. (2) Underlying data sources. Secondary analyses of clinical cohorts were eligible even when the underlying data originated from trials, provided that early adversity was not assigned and the reported analyses estimated observational exposure–outcome associations relevant to the PEO outcomes (rather than intervention effects). Because ACE exposure can accrue through age 18, studies measuring adversity during adolescence may not capture subsequent adversities up to the conventional ACE window; this may bias associations toward underestimation.

Table 2 summarises the prespecified eligibility criteria across design, population, exposure, outcomes, effect estimates, and publication features. These criteria were applied consistently during screening and full-text assessment.

### 2.4. Information Sources

Searches were conducted in PubMed, Scopus, Web of Science, ScienceDirect, SpringerLink, and Taylor & Francis. No date limits were applied. Only English-language full texts were retained, in accordance with eligibility criteria. The final search date was 1 December 2025. All sources were searched via their native platforms (PubMed, Scopus, Web of Science Core Collection, ScienceDirect, SpringerLink, and Taylor & Francis Online) from inception to 1 December 2025. No study registers, preprint servers, or other grey-literature sources were searched, and no forward/backward citation searching or author contact was undertaken to identify additional reports.

### 2.5. Search Strategy

Search concepts were built around (i) early adversity and (ii) substance-related outcomes. Because one objective was to capture evidence from psychiatric treatment/dual diagnosis settings, an additional set of psychiatric/comorbidity terms was used in a supplementary sensitivity search (run as a separate query), rather than as a mandatory constraint on the core search. Controlled vocabulary terms were mapped using Medical Subject Headings (MeSH) and Healt Sciences Descriptors (DeCS) to enhance retrieval sensitivity and harmonise concepts across databases (Coletti & Bleich, 2001; Campos et al., 2020). No date, study design, or publication status filters were applied at the search stage. Language (English) and full-text availability restrictions were applied during screening, consistent with the eligibility criteria (Table 2).

Table 3 presents the core search concepts and their controlled vocabulary mappings (DeCS/MeSH), alongside keywords and synonyms used to increase sensitivity across platforms.

### 2.6. Database-Specific Search Algorithms

The following database queries were implemented as database-specific search strings. Table 4 documents the operational search strings used in each database, including the core (early adversity × substance-related outcomes) query and the supplementary psychiatric/dual-diagnosis sensitivity query. Where interfaces differed, syntax was adapted while retaining the same conceptual structure.

### 2.7. Record Management, Screening, and Selection

All retrieved records were compiled into a central Excel database. Duplicate entries were removed prior to screening. Titles/abstracts were screened against the eligibility criteria, followed by full-text assessment. Records generating uncertainty at either stage were discussed across the review teams until consensus. Titles/abstracts and full-text reports were screened in duplicate (one reviewer from each independent review team); disagreements were resolved through discussion and full-team consensus. No automation tools were used in the selection process.

A screening log was maintained to track the following: (i) document-type exclusions, (ii) inaccessible full texts, (iii) exclusions due to ineligible operational definitions, and (iv) records retained for full-text assessment. A PRISMA flow diagram was prepared from this log (Page et al., 2021).

Table 5 reports the database-level yield log used to construct the PRISMA flow. Overlap across databases was expected; the final included set was determined after deduplication and full-text confirmation. For each database, yield counts reflect the combined export from the core and supplementary psychiatric/dual diagnosis queries shown in Table 4.

### 2.8. Data Extraction (Narrative Synthesis and Meta-Analysis Readiness)

Data were collected from each included report in duplicate: one reviewer extracted data and a second reviewer independently verified the extraction against the full text; discrepancies were resolved through discussion and full-team consensus. All included reports were in English and required no translation. Study investigators were not contacted to obtain or confirm data, and no automation tools were used for data collection.

A structured extraction framework was applied in Excel to capture, at minimum, the following: study design; setting; sample characteristics; population frame (psychiatric care vs. community); exposure operationalisation (instrument, timing, thresholds); outcome definitions (SUD/addiction vs. overdose); analytic model (adjusted vs. unadjusted); effect size type effect measures extracted included odds ratios (OR), risk ratios (RR), and hazard ratios (HR); confidence intervals and *p*-values; covariates; subgroup definitions. When information was missing or unclear in the source report (e.g., analytic sample size for a model, covariate set details, or precision measures), it was recorded as not reported (NR), and no imputation was undertaken; derived quantities were computed only when sufficient information was available.

When multiple eligible estimates were available, extraction prioritised (i) the estimate most closely aligned with the primary outcome definition, (ii) adjusted models over unadjusted models when covariate selection was clinically/epidemiologically justified, and (iii) the broadest, interpretable exposure contrast to support comparability across studies (e.g., any ACE vs. none; high vs. low burden), while preserving the original reporting. All results that were compatible with each outcome domain in each study were sought; when multiple estimates were reported within an outcome domain, one estimate per study per outcome was selected using the prespecified decision rules to support structured synthesis and, where feasible, pooling.

### 2.9. Risk of Bias Assessment (Jbi Critical Appraisal Tools)

Risk of bias assessments were performed in duplicate (one reviewer from each independent review team) using the relevant Joanna Briggs Institute (JBI) checklist for the study design; disagreements were resolved through discussion and consensus. No automation tools were used for risk of bias assessment.

Risk of bias was assessed at the study level using Joanna Briggs Institute critical appraisal tools selected by design. The revised JBI tool for cohort studies (Barker et al., 2025a) and the revised JBI tool for analytical cross-sectional studies (Barker et al., 2025b) were applied where appropriate. For designs without a revised quantitative tool in the revised series, the corresponding current JBI checklist from the JBI Manual for Evidence Synthesis was used (Joanna Briggs Institute, 2020).

Each item was judged using the checklist signalling logic, with study-specific justification recorded. A global judgement (low/moderate/high risk of bias) was derived using a prespecified rule: low when no key domain was rated “No” and ≤1 item was “Unclear”; high when any key domain (exposure validity, outcome validity, confounding identification/control, or follow-up adequacy in cohorts) was rated “No” or when ≥3 items were “No/Unclear”; the remaining patterns were classified as moderate.

### 2.10. Effect Measures and Synthesis Methods

The primary synthesis targets were association estimates linking early adversity exposure to (i) substance-related disorder/addiction outcomes (primary) and (ii) non-fatal overdose episodes (secondary). Where quantitative synthesis was feasible, effect sizes were pooled on the log scale using inverse-variance methods.

A random effects model was prespecified given expected clinical and methodological diversity across populations, exposure definitions, and outcome ascertainment (DerSimonian & Laird, 1986). Statistical heterogeneity was quantified using I^2^ (Higgins et al., 2003). When at least 10 studies contributed to a pooled estimate, small-study effects were explored using funnel-plot asymmetry testing (Egger et al., 1997).

When a meta-analysis was completed, risk of bias due to missing evidence was assessed using ROB-ME (Page et al., 2023). Certainty of evidence for exposure–outcome associations was rated using GRADE guidance for prognostic factors where pooling and interpretation supported such assessment (Foroutan et al., 2020).

Effect measures extracted included odds ratios (OR, including adjusted ORs), risk ratios (RR), and hazard ratios (HR), as reported by the primary studies. For synthesis, studies were grouped by outcome domain (SUD/addiction vs. non-fatal overdose) and by commensurability of exposure and outcome parameterisation. The overdose meta-analysis was restricted a priori to studies reporting an adjusted OR for overdose modelled per 1-point increase in total ACE score; other overdose operationalisations (e.g., CTQ domain contrasts) were retained for structured synthesis but were not pooled.

For quantitative synthesis, ORs were log-transformed, and standard errors were derived from reported 95% confidence intervals on the log scale. Random-effects meta-analysis used inverse-variance weighting with the DerSimonian–Laird estimator for between-study variance (τ^2^). Statistical heterogeneity was summarised using Cochran’s Q, I^2^, and τ^2^. Given sparse evidence, uncertainty was additionally examined using a Hartung–Knapp sensitivity analysis, and robustness was assessed using leave-one-out analyses.

Planned investigations of heterogeneity (subgroup analyses or meta-regression) and quantitative small-study effect assessments were not undertaken when the number of studies contributing to a synthesis was insufficient. Meta-analytic computations were implemented in Python 3.11.2 (NumPy 1.24.0; SciPy 1.14.1), and forest plots were generated using matplotlib 3.7.5. Risk of bias due to missing evidence (reporting biases) was assessed at the synthesis level using Risk Of Bias due to Missing Evidence (ROB-ME), and certainty of evidence was appraised using Grading of Recommendations Assessment, Development and Evaluation (GRADE) guidance for prognostic factors; both assessments were performed in duplicate with consensus resolution. Study characteristics, risk-of-bias assessments, and study-level effect estimates were presented in structured tables, and meta-analytic results were displayed using forest plots.

## 3. Results

### 3.1. Study Selection

The database searches yielded 13,206 records (PubMed, n = 1112; Web of Science, n = 645; Scopus, n = 1471; ScienceDirect, n = 4543; SpringerLink, n = 4338; Taylor & Francis, n = 1097). After the removal of document-type exclusions and review/incomplete/duplicate records (n = 7311), 5895 records were screened. Of these, 1821 were excluded due to ineligible operational definitions for the exposure and/or outcomes, and 4074 reports were sought for retrieval. Full texts could not be retrieved for 4060 reports (“no access”), leaving 14 reports for full-text eligibility assessment (Table 5). Five reports were excluded after eligibility assessment, resulting in nine observational studies included in the review (n = 9).

Five full-text reports were excluded at the eligibility stage because they did not operationalise the primary/secondary outcomes in a manner consistent with the review question (i.e., disorder-level SUD/addiction outcomes and/or non-fatal overdose events with an extractable exposure–outcome association). The excluded reports and reasons are documented below.

Table 6 lists the full-text reports excluded after eligibility assessment, with the specific PEO element(s) that failed and a concise justification. This table is intended to maintain traceability between the eligibility criteria and the final included set.

**Figure 1:**
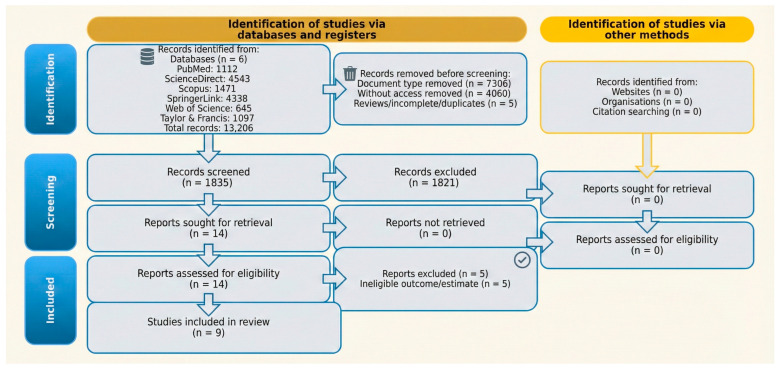
PRISMA 2020 flow diagram.

Figure 1 provides the PRISMA 2020 flow diagram summarising record management, screening, and inclusion.

### 3.2. Study Characteristics

Across the nine included studies, designs comprised six analytical cross-sectional studies and three cohort/longitudinal analyses. Study populations spanned psychiatric-treatment contexts (including dual-diagnosis and opioid treatment settings) and community-based cohorts. Early adversity was operationalised using cumulative ACE scores (continuous or categorical), specific adversity typologies (e.g., childhood sexual abuse), or validated trauma instruments (e.g., CTQ domains). Eligible outcomes included disorder-level SUD/addiction outcomes (DSM/registry-derived where reported) and/or non-fatal overdose events. The included studies were Gao et al. (2010), Lake et al. (2015), Stein et al. (2017), Bryant et al. (2020), Moss et al. (2020), McCabe et al. (2022), Tschampl et al. (2022), Broekhof et al. (2023), and Asheh et al. (2023).

Table 7 summarises the key characteristics of the included studies (design, setting, population frame, exposure and outcome operationalisation, and analytic sample size). Where the extracted text did not allow confirmation of an analytic subsample (e.g., wave-specific numbers), the corresponding field is reported as NR.

### 3.3. Risk of Bias in Included Studies

Risk of bias varied by design. Among analytical cross-sectional studies, the most frequent limitations concerned outcome ascertainment for overdose (often self-reported and not documented using objective/standard criteria) and, in some studies, uncertainty in exposure measurement related to retrospective reporting. In the cohort evidence, one registry-linkage study showed low risk of bias, while the PWID cohort exhibited several domains rated as unclear, driven mainly by incomplete reporting of follow-up completeness and handling of attrition.

Table 8 reports item-level judgements for the analytical cross-sectional studies using the JBI analytical cross-sectional checklist. Judgements are shown as Yes/No/Unclear alongside a global risk-of-bias classification derived using the prespecified rule stated in the Section 2.

Item-level judgements for the cohort/longitudinal evidence using the JBI cohort checklist are reported below, with global RoB derived using the prespecified rule.

Table 9 presents item-level judgements for the cohort/longitudinal evidence using the JBI cohort checklist, with global RoB derived using the prespecified rule. To enhance transparency, a structured risk-of-bias decision log summarising the rationale for all non-Yes (No/Unclear) judgements is provided in Table S1.

### 3.4. Results of Individual Studies

Study-level adjusted associations for the primary and secondary outcomes are presented below. Where a study reported multiple eligible estimates, we prioritised one estimate per study per outcome using the prespecified extraction rule (closest alignment with the PEO outcome definition; adjusted model where clinically/epidemiologically justified; most interpretable exposure contrast for cross-study comparability). Effect estimates are reported as published by the original studies (Gao et al., 2010; Lake et al., 2015; Stein et al., 2017; Bryant et al., 2020; Moss et al., 2020; McCabe et al., 2022; Tschampl et al., 2022; Broekhof et al., 2023; Asheh et al., 2023). Group-level summary statistics (e.g., event counts by exposure strata) were not consistently reported for the adjusted models; therefore, we tabulate adjusted effect estimates with 95% CIs and analytic sample sizes where available.

Table 10 lists the key extracted adjusted associations for the primary outcome (SUD/addiction). The selected estimate in each row is the one designated for descriptive synthesis and, where feasible, for harmonised quantitative synthesis within comparable clusters.

Table 11 presents the key extracted adjusted associations for the secondary outcome (non-fatal overdose). Three studies modelled non-fatal overdose odds per 1-point increase in ACE score, enabling a consistent exposure scaling for later quantitative synthesis; the trauma-domain model (CTQ) is retained as complementary evidence.

### 3.5. Results of Syntheses

#### 3.5.1. Quantitative Synthesis (Secondary Outcome): Non-Fatal Overdose per 1-Point Increase in ACE Score

A quantitative synthesis was feasible for the non-fatal overdose outcome because three studies reported adjusted odds ratios for lifetime/history of non-fatal overdose modelled per 1-point increase in ACE score (Stein et al., 2017; Tschampl et al., 2022; Asheh et al., 2023). Effect estimates were pooled using a random effects model (DerSimonian & Laird, 1986), with effects analysed on the log scale. Heterogeneity was low to moderate (I^2^ = 28.4%). Given the small number of contributing studies (k = 3), small-study effects were not assessed. All three contributing studies were analytical cross-sectional treatment-based samples and were rated high risk of bias, primarily due to overdose outcome ascertainment not using objective/standard criteria (Table 8 and Table S1).

Table 12 provides the effect sizes used in the overdose meta-analysis, including the published adjusted ORs with 95% CIs and the derived log (OR) and standard errors used for inverse-variance pooling.

Table 13 summarises the random-effects meta-analysis for non-fatal overdose, showing study-specific and pooled estimates. A Hartung–Knapp sensitivity analysis is reported to reflect uncertainty with k = 3.

**Figure 2:**
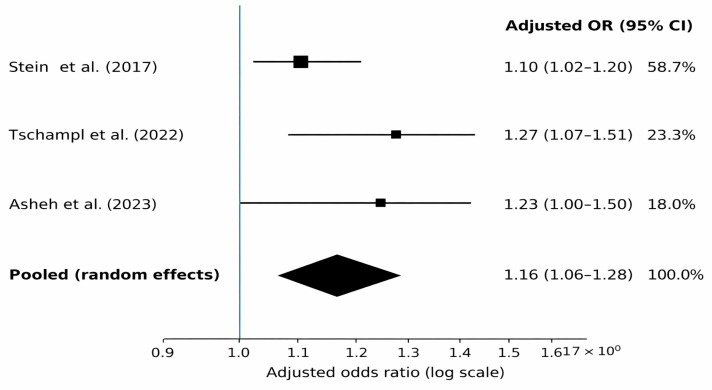
Forest plot for non-fatal overdose meta-analysis (ACE score per +1 point). Studies: Stein et al. (2017), Tschampl et al. (2022) and Asheh et al. (2023).

Figure 2 presents the forest plot for the non-fatal overdose meta-analysis based on the three contributing studies. 

#### 3.5.2. Evidence Not Pooled for Overdose (Structured Synthesis)

One cohort study assessed childhood adversity using CTQ domain scores rather than a cumulative ACE count (Lake et al., 2015). Because CTQ domain contrasts are not commensurate with “per 1 ACE point”, this evidence was retained for structured synthesis rather than combined in the quantitative model. In that study, childhood trauma domains were associated with higher odds of non-fatal overdose, including CTQ physical abuse (OR 1.36, 95% CI 1.08–1.71) (Lake et al., 2015). This cohort study was rated as having a high risk of bias because several follow-up domains were unclear and overdose outcome ascertainment relied on self-report (Table 9 and Table S1).

#### 3.5.3. Primary Outcome (SUD/Addiction): Structured Narrative Synthesis (Pooling Not Undertaken)

Five included studies contributed adjusted associations for the primary outcome (Gao et al., 2010; Bryant et al., 2020; Moss et al., 2020; McCabe et al., 2022; Broekhof et al., 2023). These studies consistently aligned early adversity with higher odds of disorder-level substance-related outcomes, but quantitative pooling was not undertaken because key elements required for commensurate synthesis differed across studies: (i) exposure scaling (specific maltreatment domains vs. cumulative ACE burden vs. CSA typologies), (ii) outcome operationalisation (any SUD diagnosis vs. severity-based disorder constructs vs. registry-derived diagnoses), and (iii) model specification (non-uniform confounder sets and subgroup stratification). Accordingly, the primary-outcome evidence was synthesised descriptively using the prioritised estimates reported in Table 10.

All five primary-outcome studies were rated as low risk of bias at the study level (Table 8, Table 9 and Table S1) but were not pooled due to non-commensurate exposure and outcome definitions.

### 3.6. Reporting Biases and Missing Evidence

Formal assessment of small-study effects (funnel-plot asymmetry and regression-based tests) was not undertaken for any synthesis because no pooled analysis included ≥10 studies (Egger et al., 1997). For the overdose meta-analysis (k = 3), the risk of bias due to missing evidence was appraised at the synthesis level using ROB-ME, acknowledging that quantitative indicators of dissemination bias are not informative with such sparse evidence (Page et al., 2023).

Table 14 summarises the ROB-ME judgement for the overdose meta-analysis, documenting the main considerations relevant to missing evidence within the constraints of the available corpus and the review’s eligibility restrictions (e.g., English-language full texts).

### 3.7. Certainty of Evidence

Certainty in the evidence for prognostic-factor style associations was appraised using GRADE guidance for prognostic factors, where observational bodies of evidence may be rated down based on risk of bias, imprecision, inconsistency, indirectness, and publication bias (Foroutan et al., 2020). The certainty assessment was prioritised for the quantitative synthesis (overdose) since this outcome produced a pooled estimate.

Table 15 presents a summary of the findings for the overdose meta-analysis, including the pooled relative association and the rationale for rating decisions across core GRADE domains.

### 3.8. Additional Analyses

Two robustness checks were applied to the overdose meta-analysis, given the small number of contributing studies (k = 3). First, a Hartung–Knapp adjustment was used to evaluate the sensitivity of uncertainty estimates in random effects models with sparse evidence (Hartung & Knapp, 2001; IntHout et al., 2014). Second, a leave-one-out analysis examined whether the pooled effect was disproportionately driven by any single study. Subgroup analyses, meta-regression, and formal small-study effect testing were not performed because the number of studies was insufficient for defensible inference (Egger et al., 1997).

Table 16 reports the results of the leave-one-out analysis for the non-fatal overdose synthesis, alongside the Hartung–Knapp sensitivity estimate for comparison with the DerSimonian–Laird random effects result presented in Section 3.5.

### 3.9. Direction and Consistency of Associations Across Outcomes

Across the included studies, the direction of association was consistently positive (OR/aOR > 1) for both the primary (SUD/addiction) and secondary (overdose) outcomes when adversity exposure was higher, although exposure scaling and outcome operationalisation varied materially across studies (Gao et al., 2010; Lake et al., 2015; Stein et al., 2017; Bryant et al., 2020; Moss et al., 2020; McCabe et al., 2022; Tschampl et al., 2022; Broekhof et al., 2023; Asheh et al., 2023). For overdose, three studies reported commensurate per 1-point ACE estimates (Stein et al., 2017; Tschampl et al., 2022; Asheh et al., 2023), whereas one cohort used CTQ trauma domains and was retained outside the pooled model (Lake et al., 2015). For SUD/addiction, selected study-level estimates spanned different constructs (any SUD, substance-specific disorder, severity-based endpoints, and registry-derived diagnoses), limiting comparability despite consistent directionality (Gao et al., 2010; Bryant et al., 2020; Moss et al., 2020; McCabe et al., 2022; Broekhof et al., 2023).

Table 17 summarises the direction of associations using the prespecified, prioritised estimates (Table 10 and Table 11) and distinguishes between the pooled overdose synthesis (ACE per +1 point) and non-pooled evidence retained for structured synthesis.

## 4. Discussion

### 4.1. Interpretation in Relation to the Review Question

Across nine observational studies spanning psychiatric treatment settings and community samples, early adverse experiences were consistently associated with increased odds of substance-related disorder (SUD) outcomes and/or overdose events. The direction of association was aligned across heterogeneous operationalisations of exposure (single-domain maltreatment history, cumulative ACE scores, CTQ domains, and CSA typologies) and across outcome ascertainment approaches (clinical diagnoses, registry linkage, survey-derived DSM-5 outcomes, and self-reported overdose history). The consistency in direction across settings matters for the PEO framing used here because it reduces the likelihood that the association is confined to a single clinical context or a single measurement instrument.

At the same time, the evidence base is not uniform. Treatment-based samples (including opioid detoxification, outpatient addiction care, and dual-diagnosis services) largely capture individuals with substantial clinical complexity, where adversity exposure may co-occur with psychiatric comorbidity, socioeconomic disadvantage, and other determinants that are not measured identically across studies (Stein et al., 2017; Asheh et al., 2023; Tschampl et al., 2022). Community cohorts with prospective follow-up and registry linkage offer stronger temporal alignment between exposure and later diagnosis yet still rely on retrospective adversity measurement in many cases (Broekhof et al., 2023). In this review, the collective pattern is compatible with ACEs operating as a risk marker for later SUD morbidity and overdose vulnerability rather than as a single sufficient cause.

#### 4.1.1. Quantitative Synthesis for Non-Fatal Overdose (ACE Score per +1 Point)

Quantitative pooling was defensible only for the secondary outcome (overdose) under a single exposure parameterisation: adjusted odds ratios per one-point increase in ACE score. Three studies contributed to this synthesis (Stein et al., 2017; Tschampl et al., 2022; Asheh et al., 2023), yielding a random effects pooled estimate of OR 1.16 (95% CI 1.06–1.28) with low-to-moderate heterogeneity (I^2^ ≈ 28%). The pooled point estimate implies that incremental increases in cumulative adversity burden carry measurable differences in overdose odds within the studied populations, even when models adjust for covariates selected by the original authors.

Uncertainty remains material. With k = 3, a Hartung–Knapp sensitivity approach produced a wider interval that crossed the null (OR 1.16, 95% CI 0.95–1.41). Under these conditions, inference benefits from attention to the magnitude and stability of the point estimate across modelling approaches rather than reliance on threshold-based declarations. Small-study effects were not assessed because the conventional minimum for funnel-plot-based asymmetry tests was not met.

Lake et al. (2015) reinforced the overdose link using a different exposure framework (CTQ abuse domains rather than ACE count) and reported positive associations with non-fatal overdose for multiple trauma domains. This evidence complements the pooled estimate but was not combined in the same model because CTQ subscale contrasts are not commensurate with a one-unit ACE increment.

#### 4.1.2. Why the Primary Outcome Meta-Analysis Was Constrained

Pooling for SUD/addiction outcomes was not methodologically defensible without stronger harmonisation because studies diverged on both the exposure scale and the outcome definition. Exposure metrics ranged from binary indicators of childhood physical or sexual abuse (Gao et al., 2010), dose patterns across ACE categories (Bryant et al., 2020), and CSA type counts (McCabe et al., 2022), to “any ACE” and accumulated ACE scores in a linked cohort (Broekhof et al., 2023), alongside ACE burden, which was linked to severe substance-specific outcomes in a longitudinal dataset (Moss et al., 2020). Outcome definitions also varied: “any SUD” diagnoses in service settings, substance-specific dependence or severity thresholds, and registry-confirmed diagnoses. Combining these estimates would have blended meaningfully different constructs (both on the exposure side and on the clinical endpoint side), risking a pooled value that is difficult to interpret and potentially misleading.

This constraint is not unique to this review. Prior syntheses have highlighted that ACE research often faces conceptual and measurement heterogeneity despite strong associations across multiple adult health outcomes (Hughes et al., 2017; Madigan et al., 2023). For opioid-related outcomes specifically, recent reviews have reported robust links between childhood adversity and opioid use-related behaviours while noting variability in outcome definitions and analytic adjustment (Meyer et al., 2023; Regmi et al., 2024). This present review encountered the same structural issue at the point of meta-analytic decision-making: the evidence is informative but only some parts are sufficiently commensurable for pooling.

### 4.2. Implications for Certainty, Practice, and Evidence Synthesis

#### 4.2.1. Clinical and Service Implications

The observed associations carry implications for clinical assessment pathways in psychiatric and addiction services. In treatment settings, elevated ACE burden may mark individuals who require integrated approaches that address trauma exposure alongside substance-related risk management, including overdose prevention strategies. At the same time, routine ACE screening is contested when implemented without adequate downstream capacity, clear referral pathways, and trauma-informed safeguards (Gentry & Paterson, 2022; Racine et al., 2020). For practice, the evidence favours a stance in which adversity history informs formulation and risk stratification within a trauma-informed model, rather than functioning as a stand-alone screening exercise detached from intervention resources.

#### 4.2.2. Implications for the Evidence Base

Progress in this area depends less on producing additional isolated associations and more on improving commensurability. Comparability would increase through (i) agreed exposure contrasts (for example, per-unit ACE increment and a common high vs. none threshold reported side-by-side), (ii) clearer outcome harmonisation (distinguishing incident SUD diagnoses from severity strata and separating overdose endpoints), (iii) transparent covariate sets motivated by explicit causal reasoning, and (iv) prospective designs where feasible, particularly for incident SUD outcomes in community cohorts. Within treatment samples, standardised reporting of overdose definition (timeframe, ascertainment source, and intentionality) would also reduce ambiguity.

In this present review, the most defensible quantitative statement concerns overdose risk under a shared ACE score increment model, supported by low-to-moderate heterogeneity and a stable pooled point estimate under alternative uncertainty estimation. For SUD outcomes, the evidence is persuasive in direction across diverse settings, yet synthesis is best treated as a structured narrative unless a pre-specified harmonisation rule is applied and consistently extractable estimates are available across studies.

#### 4.2.3. Implications for Clinical Services and Public Health (Practice-Facing Synthesis)

Across the included clinical and community studies, higher exposure to early adverse experiences was associated with higher odds of substance-related outcomes and overdose, with effect estimates that were directionally consistent despite heterogeneity in measurement and case definition. In practical terms, the pooled overdose estimate (OR 1.16 per 1-point ACE increase) represents an incremental risk gradient rather than a deterministic marker; implementation in services is best framed around risk stratification and linkage to supports, not prediction at the individual level.

Within psychiatric and addiction care pathways, these patterns align with the use of trauma-informed service design that reduces re-traumatisation, improves engagement, and integrates mental health and substance use care. Even when the ACE history is not formally quantified, clinical decision-making can incorporate a structured inquiry about adversity in a way that prioritises safety, choice, collaboration, and cultural humility. When programmes elect to adopt ACE screening tools, they should avoid treating screening as a “stand-alone” intervention; benefits depend on downstream capacity (brief intervention, referral options, safeguarding workflows, and staff training), and screening can be ethically problematic when services cannot respond. Evidence syntheses on ACE screening highlight recurring concerns about readiness, criteria for screening programmes, and potential harm when systems are not prepared to act on disclosures (Gentry & Paterson, 2022; Racine et al., 2020).

At a public health level, the observational nature of the evidence base precludes causal claims in this review, but it remains compatible with prevention approaches that reduce childhood adversity exposure and strengthen protective environments. A high prevalence of ACE exposure in adult populations has been documented in large meta-analytic work, which provides context for the scale of potential downstream burden (Madigan et al., 2023). For policy, the implication is not that ACEs “explain” opioid- or substance-related epidemics but that adversity-informed prevention and care pathways are coherent with the epidemiology and with the overdose signal observed in the quantitative synthesis.

### 4.3. Research Priorities for a More Poolable Evidence Base (Method-Facing Synthesis)

Quantitative synthesis for the non-fatal overdose outcome was feasible only within a narrow harmonisation window (ACE score modelled per 1-point increase), and the Hartung–Knapp sensitivity analysis widened uncertainty as expected with small k. For the primary outcome (SUD/addiction), pooling remained methodologically fragile because studies differed simultaneously in (i) adversity operationalisation (single-domain maltreatment vs. cumulative ACE burden vs. CSA typologies), (ii) outcome definition (any SUD vs. substance-specific diagnoses vs. severity thresholds; registry vs. self-report), and (iii) adjustment sets and subgroup stratification. Progress towards a more poolable literature base depends on three converging improvements: Shared exposure contrasts: routine reporting of both a continuous ACE score effect (per-point) and a categorical contrast (e.g., ≥4 vs. 0), alongside clear timing of measurement and handling of missing ACE items. Outcome harmonisation: explicit mapping to DSM/ICD definitions for SUD where diagnosis is the target, as well as standardised overdose definitions (non-fatal vs. fatal; timeframe; ascertainment method). Transparent confounding strategy: consistent reporting of the confounder set, rationale for inclusion, and sensitivity checks addressing residual confounding, measurement error, and differential misclassification. In the Hartung–Knapp sensitivity analysis, the 95% CI crossed the null (0.95–1.41), indicating non-significance under that approach.

Recent systematic reviews outside the included set reinforce that ACE–opioid associations are repeatedly observed across heterogeneous designs, while also documenting the same measurement and comparability barriers that limit meta-analytic aggregation (Meyer et al., 2023; Regmi et al., 2024; Deol et al., 2023). Aligning future primary studies to a minimal reporting core would materially improve synthesis feasibility without constraining substantive innovation.

### 4.4. Closing Statement (Interpretive Boundary Aligned to the Design)

In adult psychiatric treatment and community samples, early adverse experiences were associated with higher odds of substance-related outcomes, and the available poolable evidence for overdose supports a small-to-moderate incremental risk gradient per ACE point. Interpretation is bounded by observational designs and heterogeneity in exposure and outcome measurement. The most defensible synthesis strategy in this review remains structured narrative integration across all studies, paired with quantitative pooling restricted to commensurate definitions.

### 4.5. Strengths

This review was conducted using a structured, reproducible workflow aligned with PRISMA 2020 and PRISMA-S, with explicit a priori eligibility anchored to a PEO aetiological question and documented screening decisions (Page et al., 2021; Rethlefsen et al., 2021). Study-level risk of bias was appraised using design-appropriate JBI tools, and quantitative synthesis was restricted to exposure–outcome parameterisations that were commensurate (DerSimonian & Laird, 1986). For the overdose outcome, pooling per one-point increase in ACE score reduced avoidable incompatibility across studies while allowing for heterogeneity to be quantified and interpreted (Higgins et al., 2003).

### 4.6. Implications for Clinical Services and Public Health

The overdose synthesis indicates a graded association between cumulative adversity burden and overdose history when the ACE score is modelled per 1-point increase (Stein et al., 2017; Tschampl et al., 2022; Asheh et al., 2023). In service contexts, this pattern supports the use of adversity history as a risk marker within integrated assessment, particularly where overdose prevention is already part of care pathways. The magnitude is clinically interpretable as incremental rather than deterministic, and it fits models of care that combine psychiatric assessment, substance-use treatment, and harm-reduction components rather than treating adversity exposure as an isolated screening endpoint.

Implementation remains contingent on system readiness. Evidence-based critiques of routine ACE screening emphasise that inquiry about adversity is ethically and clinically defensible only when accompanied by trauma-informed practice, staff training, and clear response capacity (Racine et al., 2020; Gentry & Paterson, 2022). In settings with constrained referral options or limited safeguarding infrastructure, the routine quantification of ACEs can generate disclosures without meaningful support. In those contexts, a formulation-led, trauma-informed approach—centred on safety, choice, collaboration, and avoidance of re-traumatisation—better matches the evidential limits of observational associations (Racine et al., 2020).

At a public health level, the present evidence does not justify causal claims, yet it aligns with broader epidemiological work showing that ACE exposure is common and associated with a wide range of adverse adult health outcomes (Hughes et al., 2017; Madigan et al., 2023). Prevention strategies that reduce childhood adversity exposure and strengthen protective environments remain coherent with this burden, while clinical systems benefit from integrating adversity-informed assessment with overdose prevention and mental health/substance use comorbidity care.

#### Implications for Research and Evidence Synthesis

Future research will be most valuable if it increases commensurability across studies without flattening substantive complexity. For quantitative synthesis, this present review shows that pooling becomes feasible when the exposure metric and outcome definition are aligned—illustrated by the overdose meta-analysis restricted to adjusted ORs per 1-point increase in ACE score (DerSimonian & Laird, 1986; Higgins et al., 2003). By contrast, the SUD/addiction evidence base remained non-poolable because exposure operationalisations (single-domain maltreatment, cumulative ACE burden, CSA typologies, CTQ subscales) and outcome definitions (any SUD, substance-specific disorders, severity thresholds, registry diagnoses) varied simultaneously. Standardised reporting would materially increase synthesis efficiency and interpretability.

Four priorities follow. First, studies should routinely report paired ACE contrasts (continuous per-point effect and a pre-specified categorical threshold such as ≥4 vs. 0), alongside the transparent handling of missing ACE items. Second, SUD outcomes should be reported with explicit DSM/ICD mapping and timeframes, while overdose definitions should specify fatal/non-fatal status, the ascertainment method (self-report, clinical record, registry), and the reference period. Third, confounding strategies should be documented with clear rationale and sensitivity checks for residual confounding and misclassification. Fourth, where feasible, prospective designs and registry linkage can strengthen temporal plausibility and reduce outcome misclassification while still requiring careful attention to exposure measurement quality.

These priorities are consistent with recurring conclusions in related ACE synthesis work, where strong associations are observed across adult outcomes, but meta-analytic aggregation is frequently constrained by measurement heterogeneity and inconsistent reporting (Hughes et al., 2017; Madigan et al., 2023). For opioid-related outcomes, recent systematic reviews report similar barriers while confirming that childhood adversity is repeatedly linked to opioid use-related behaviours (Meyer et al., 2023; Regmi et al., 2024). The implication for evidence synthesis is that future primary studies should treat reporting choices as infrastructure for cumulative science: the goal is not only internal validity within a single dataset but interpretability across studies.

### 4.7. Limitations

Several limitations constrain inference. First, the evidence base is observational and heterogeneous: most studies are cross-sectional or retrospective, limiting temporality, and adjustment sets varied, leaving scope for residual confounding. Second, exposure and outcome operationalisation differed markedly (e.g., ACE score versus domain-specific trauma measures; ICD-coded SUD versus self-report), which constrained meta-analysis without imposing assumptions that would compromise commensurability. Third, the non-fatal overdose meta-analysis was based on k = 3 studies; pooled estimates were method-sensitive (e.g., Hartung–Knapp) and should be interpreted cautiously.

Regarding overdose, the included studies ascertained overdose among living participants and therefore reflect non-fatal overdose history (often self-reported). Non-fatal overdoses are several times more common than fatal overdoses (World Health Organization, 2025), and non-fatal overdose has been associated with an increased subsequent risk of fatal overdose among people who inject drugs (Caudarella et al., 2016). We did not identify eligible studies reporting fatal overdose outcomes. If the relationship between early adversity and substance-related pathology extends along a continuum, excluding fatal overdose may underestimate associations at the most severe end of the spectrum. This highlights a priority for future studies to report fatal and non-fatal overdose separately and to specify ascertainment (self-report versus clinical or administrative records).

Age-related measurement may also contribute to conservative estimates. Although we excluded studies restricted to participants < 18 years, some included cohorts recruited adolescents and measured adversity at baseline. Because adversity can accrue through age 18, such designs may not capture exposures occurring after baseline measurement, potentially attenuating associations.

Finally, a substantial proportion of records could not be retrieved in full text (“no access”), and only English-language full texts were retained; these constraints may introduce selection and publication biases. In addition, the review did not search study registers, preprint servers, or other grey-literature sources, and we did not conduct forward/backward citation searching or contact study authors to identify additional reports. These review process limitations may have increased the risk of missing relevant studies.

## 5. Conclusions

In adult psychiatric treatment and community populations, early adverse experiences were associated with higher odds of substance-related outcomes across the included observational studies, with consistent directionality despite variation in exposure and outcome definitions (Gao et al., 2010; Bryant et al., 2020; Moss et al., 2020; McCabe et al., 2022; Broekhof et al., 2023; Lake et al., 2015; Stein et al., 2017; Tschampl et al., 2022; Asheh et al., 2023). Quantitative synthesis was defensible only for non-fatal overdose when the exposure was harmonised as an ACE score per 1-point increase, yielding a random effect pooled estimate of OR 1.16 (95% CI 1.06–1.28). In the Hartung–Knapp sensitivity analysis, the 95% CI crossed the null (OR 1.16, 95% CI 0.95–1.41), indicating non-significance under that more conservative approach when k = 3 (Hartung & Knapp, 2001; IntHout et al., 2014). For the primary SUD/addiction outcome, heterogeneity in operational definitions and modelling choices constrained meta-analysis without imposing assumptions that would reduce interpretability.

From a practice perspective, adversity burden functions as a risk marker that can provide important perspectives for trauma-informed assessment and non-fatal overdose prevention pathways, provided inquiry is embedded within service capacity and safeguarding workflows rather than implemented as stand-alone screening (Racine et al., 2020; Gentry & Paterson, 2022). From an evidence synthesis perspective, commensurability would improve through consistent reporting of paired exposure contrasts, explicit diagnostic mapping for SUD outcomes, and transparent confounding strategies, enabling future syntheses to combine estimates without mixing non-equivalent constructs (Hughes et al., 2017; Madigan et al., 2023). A concise proposed reporting core that supports commensurability is summarised in Table 18.

## Figures and Tables

**Table 1:** Research question framed using PEO.

Acronym	Element	Description
P	Population	Psychiatric patients receiving psychological, psychiatric, or integrated care, or individuals living in the community
E	Exposure	Early adverse experiences (adverse childhood experiences/childhood adversity/childhood trauma and related constructs)
O (primary)	Outcome	Probability of developing substance-related disorders/addictions
O (secondary)	Outcome	Probability of experiencing at least one non-fatal overdose episode

Notes. PEO, Population–Exposure–Outcome. “Early adverse experiences” includes ACE-type cumulative measures and specific adversity constructs (e.g., abuse, neglect, household dysfunction) when explicitly measured.

**Table 2:** Eligibility criteria.

Domain	Inclusion Criteria	Exclusion Criteria
Study design	Observational studies: cohort, case–control, analytical cross-sectional	Reviews (systematic/narrative/meta-analysis), editorials, conference abstracts, letters, protocols without results; randomised trials and other experimental designs (intervention-effect analyses); ecological studies; qualitative studies; case reports/series
Population	Psychiatric patients in psychological/psychiatric/integrated treatment or community-based populations	Populations restricted to minors with outcomes measured in childhood/adolescence; incarcerated populations
Exposure	Explicit assessment of early adverse experiences (e.g., ACEs, childhood trauma/adversity, maltreatment/abuse/neglect)	Exposure not representing an early adversity construct
Outcomes	At least one eligible outcome: substance-related disorder/addiction outcome (primary) and/or non-fatal overdose episode (secondary)	Studies reporting other psychiatric outcomes without an exposure–outcome association for the eligible outcomes
Effect estimate	Reports an association/risk measure (e.g., OR, RR, HR) for at least one eligible outcome	No extractable association estimate linking exposure to an eligible outcome
Publication features	Full text available; English language; no date restrictions	Non-English full text; inaccessible full text

Notes. OR, odds ratio; RR, risk ratio; HR, hazard ratio. Eligibility focused on extractable exposure–outcome associations for the primary and/or secondary outcomes defined in Table 1.

**Table 3:** Search concepts, controlled vocabulary, and synonyms.

Concept	Main Terms	DeCS	MeSH	Additional Synonyms
Adverse Childhood Experiences	Adverse Childhood Experiences	Experiencias Adversas en la Infancia	Adverse Childhood Experiences	Early life stress; Childhood trauma; Traumatic childhood experiences
Substance-Related Disorders	Substance-Related Disorders	Trastornos Relacionados con Sustancias	Substance-Related Disorders	Drug abuse; Substance abuse; Substance dependence; Chemical dependency
Addiction disorders	Addiction disorders	Trastornos por Adicción	Substance Use Disorders	Drug and Alcohol Use Disorders
Psychiatric/dual-diagnosis context (supplementary sensitivity block)	Dual Diagnosis Patients	Trastornos Mentales Comórbidos	Dual Diagnosis	Co-occurring disorders; Patients with co-occurring disorders

Notes. DeCS, Health Sciences Descriptors; MeSH, Medical Subject Headings. Terms were adapted to database-specific syntax and field tags.

**Table 4:** Search algorithms by database (core and supplementary psychiatric/dual-diagnosis queries).

Database	Search Equation
PubMed	Core query: (“Adverse Childhood Experiences” OR ACEs OR “Childhood trauma*” OR “Childhood adversity*” OR “Early life stress*” OR “Early trauma*” OR “Child abuse” OR “Child neglect” OR “Parental neglect” OR “Child maltreatment” OR “Early adverse experience*”) AND (“Substance Use Disorder*” OR “Substance-Related Disorder*” OR “Substance abuse” OR “Substance dependence” OR “Drug use disorder*” OR “Drug abuse” OR “Alcohol use disorder*” OR “Alcohol abuse” OR “Addiction*” OR “Chemical dependence”) Supplementary psychiatric/dual-diagnosis query: (“Adverse Childhood Experiences” OR ACEs OR “Childhood trauma*” OR “Childhood adversity*” OR “Early life stress*” OR “Early trauma*” OR “Child abuse” OR “Child neglect” OR “Parental neglect” OR “Child maltreatment” OR “Early adverse experience*”) AND (“Substance Use Disorder*” OR “Substance-Related Disorder*” OR “Substance abuse” OR “Substance dependence” OR “Drug use disorder*” OR “Drug abuse” OR “Alcohol use disorder*” OR “Alcohol abuse” OR “Addiction*” OR “Chemical dependence”) AND (“Dual diagnosis” OR “Co-occurring disorder*” OR “Comorbid disorder*” OR “Psychiatric disorder*” OR “Mental disorder*” OR “Psychiatric patient*” OR “Severe mental illness” OR SMI)
Scopus	Core query: TITLE-ABS-KEY((“Adverse Childhood Experiences” OR ACEs OR “Childhood trauma*” OR “Childhood adversity*” OR “Early life stress*” OR “Early trauma*” OR “Child abuse” OR “Child neglect” OR “Parental neglect” OR “Child maltreatment” OR “Early adverse experience*”) AND (“Substance Use Disorder*” OR “Substance-Related Disorder*” OR “Substance abuse” OR “Substance dependence” OR “Drug use disorder*” OR “Drug abuse” OR “Alcohol use disorder*” OR “Alcohol abuse” OR “Addiction*” OR “Chemical dependence”)) Supplementary psychiatric/dual-diagnosis query: TITLE-ABS-KEY((“Adverse Childhood Experiences” OR ACEs OR “Childhood trauma*” OR “Childhood adversity*” OR “Early life stress*” OR “Early trauma*” OR “Child abuse” OR “Child neglect” OR “Parental neglect” OR “Child maltreatment” OR “Early adverse experience*”) AND (“Substance Use Disorder*” OR “Substance-Related Disorder*” OR “Substance abuse” OR “Substance dependence” OR “Drug use disorder*” OR “Drug abuse” OR “Alcohol use disorder*” OR “Alcohol abuse” OR “Addiction*” OR “Chemical dependence”) AND (“Dual diagnosis” OR “Co-occurring disorder*” OR “Comorbid disorder*” OR “Psychiatric disorder*” OR “Mental disorder*” OR “Psychiatric patient*” OR “Severe mental illness” OR SMI))
Web of Science	Core query: TS = ((“Adverse Childhood Experiences” OR ACEs OR “Childhood trauma*” OR “Childhood adversity*” OR “Early life stress*” OR “Early trauma*” OR “Child abuse” OR “Child neglect” OR “Parental neglect” OR “Child maltreatment” OR “Early adverse experience*”) AND (“Substance Use Disorder*” OR “Substance-Related Disorder*” OR “Substance abuse” OR “Substance dependence” OR “Drug use disorder*” OR “Drug abuse” OR “Alcohol use disorder*” OR “Alcohol abuse” OR “Addiction*” OR “Chemical dependence”)) Supplementary psychiatric/dual-diagnosis query: TS = ((“Adverse Childhood Experiences” OR ACEs OR “Childhood trauma*” OR “Childhood adversity*” OR “Early life stress*” OR “Early trauma*” OR “Child abuse” OR “Child neglect” OR “Parental neglect” OR “Child maltreatment” OR “Early adverse experience*”) AND (“Substance Use Disorder*” OR “Substance-Related Disorder*” OR “Substance abuse” OR “Substance dependence” OR “Drug use disorder*” OR “Drug abuse” OR “Alcohol use disorder*” OR “Alcohol abuse” OR “Addiction*” OR “Chemical dependence”) AND (“Dual diagnosis” OR “Co-occurring disorder*” OR “Comorbid disorder*” OR “Psychiatric disorder*” OR “Mental disorder*” OR “Psychiatric patient*” OR “Severe mental illness” OR SMI))
Taylor & Francis	Core query: (“Adverse Childhood Experiences” OR ACEs OR “Childhood trauma*” OR “Childhood adversity*” OR “Early life stress*” OR “Early trauma*” OR “Child abuse” OR “Child neglect” OR “Parental neglect” OR “Child maltreatment” OR “Early adverse experience*”) AND (“Substance Use Disorder*” OR “Substance-Related Disorder*” OR “Substance abuse” OR “Substance dependence” OR “Drug use disorder*” OR “Drug abuse” OR “Alcohol use disorder*” OR “Alcohol abuse” OR “Addiction*” OR “Chemical dependence”) Supplementary psychiatric/dual-diagnosis query: (“Adverse Childhood Experiences” OR ACEs OR “Childhood trauma*” OR “Childhood adversity*” OR “Early life stress*” OR “Early trauma*” OR “Child abuse” OR “Child neglect” OR “Parental neglect” OR “Child maltreatment” OR “Early adverse experience*”) AND (“Substance Use Disorder*” OR “Substance-Related Disorder*” OR “Substance abuse” OR “Substance dependence” OR “Drug use disorder*” OR “Drug abuse” OR “Alcohol use disorder*” OR “Alcohol abuse” OR “Addiction*” OR “Chemical dependence”) AND (“Dual diagnosis” OR “Co-occurring disorder*” OR “Comorbid disorder*” OR “Psychiatric disorder*” OR “Mental disorder*” OR “Psychiatric patient*” OR “Severe mental illness” OR SMI)
SpringerLink	Core query: (“Adverse Childhood Experiences” OR ACEs OR “Childhood trauma*” OR “Childhood adversity*” OR “Early life stress*” OR “Early trauma*” OR “Child abuse” OR “Child neglect” OR “Parental neglect” OR “Child maltreatment” OR “Early adverse experience*”) AND (“Substance Use Disorder*” OR “Substance-Related Disorder*” OR “Substance abuse” OR “Substance dependence” OR “Drug use disorder*” OR “Drug abuse” OR “Alcohol use disorder*” OR “Alcohol abuse” OR “Addiction*” OR “Chemical dependence”) Supplementary psychiatric/dual-diagnosis query: (“Adverse Childhood Experiences” OR ACEs OR “Childhood trauma*” OR “Childhood adversity*” OR “Early life stress*” OR “Early trauma*” OR “Child abuse” OR “Child neglect” OR “Parental neglect” OR “Child maltreatment” OR “Early adverse experience*”) AND (“Substance Use Disorder*” OR “Substance-Related Disorder*” OR “Substance abuse” OR “Substance dependence” OR “Drug use disorder*” OR “Drug abuse” OR “Alcohol use disorder*” OR “Alcohol abuse” OR “Addiction*” OR “Chemical dependence”) AND (“Dual diagnosis” OR “Co-occurring disorder*” OR “Comorbid disorder*” OR “Psychiatric disorder*” OR “Mental disorder*” OR “Psychiatric patient*” OR “Severe mental illness” OR SMI)
ScienceDirect	Core query: (title, abstract, keywords): (“Adverse Childhood Experiences” OR ACEs OR “Childhood trauma*” OR “Childhood adversity*” OR “Early life stress*” OR “Early trauma*” OR “Child abuse” OR “Child neglect” OR “Parental neglect” OR “Child maltreatment” OR “Early adverse experience*”) AND (“Substance Use Disorder*” OR “Substance-Related Disorder*” OR “Substance abuse” OR “Substance dependence” OR “Drug use disorder*” OR “Drug abuse” OR “Alcohol use disorder*” OR “Alcohol abuse” OR “Addiction*” OR “Chemical dependence”) Supplementary psychiatric/dual-diagnosis query: (title, abstract, keywords): (“Adverse Childhood Experiences” OR ACEs OR “Childhood trauma*” OR “Childhood adversity*” OR “Early life stress*” OR “Early trauma*” OR “Child abuse” OR “Child neglect” OR “Parental neglect” OR “Child maltreatment” OR “Early adverse experience*”) AND (“Substance Use Disorder*” OR “Substance-Related Disorder*” OR “Substance abuse” OR “Substance dependence” OR “Drug use disorder*” OR “Drug abuse” OR “Alcohol use disorder*” OR “Alcohol abuse” OR “Addiction*” OR “Chemical dependence”) AND (“Dual diagnosis” OR “Co-occurring disorder*” OR “Comorbid disorder*” OR “Psychiatric disorder*” OR “Mental disorder*” OR “Psychiatric patient*” OR “Severe mental illness” OR SMI)

Notes. Database interfaces vary in field tags and indexing; strings were implemented to preserve conceptual equivalence. Where required by platform constraints, truncation and phrase searching were adjusted without changing the two-domain core logic (early adversity × substance-related outcomes) and the supplementary three-domain sensitivity logic (adding psychiatric/dual-diagnosis terms). The asterisk (*) is used as a truncation operator to retrieve all term variants beginning with the same root.

**Table 5:** Search yield log (database-level screening overview).

Database	Total Retrieved	Document Type	No Access	Reviews/Incomplete/Duplicates	Ineligible Variable Criteria	Total Sample
PubMed	1112	338	655	2	108	9
Web of Science	645	555	37	2	49	2
Scopus	1471	382	627	1	460	1
ScienceDirect	4543	3639	704	0	198	2
SpringerLink	4338	1954	1539	0	845	0
Taylor & Francis	1097	438	498	0	161	0
Total	13,206	7306	4060	5	1821	14

Notes. “Total sample” reflects the number of records retained after database-level filtering within the screening log. Database overlap was expected; the final included set was determined after deduplication and full-text eligibility confirmation. “No access” denotes reports for which full texts could not be retrieved (e.g., paywalled or otherwise unavailable) via available institutional subscriptions or open-access sources during screening.

**Table 6:** Full-text reports excluded after assessment (n = 5).

Study	Primary Reason for Exclusion	PEO Element Not Met	Brief Justification
Banducci et al. (2014)	Ineligible outcome	O	Outcomes focused on behavioural/psychosocial endpoints rather than SUD/addiction diagnoses or overdose events.
Vivalya et al. (2023)	Ineligible outcome/non-aligned association	O	Outcomes centred on psychiatric diagnostic context without a clearly eligible SUD/addiction or overdose exposure–outcome estimate aligned with the review’s outcome definitions.
Cunradi et al. (2020)	Outcome not at disorder/overdose level	O	Substance outcomes were risk/use indicators (e.g., hazardous use patterns) rather than disorder-level SUD/addiction outcomes or overdose events.
Kascakova et al. (2022)	Outcome operationalisation not eligible for this synthesis	O	Substance outcomes relied on screening-based indicators rather than disorder-level diagnoses/registry outcomes or overdose events, limiting comparability with the primary outcome definition.
al’Absi et al. (2023)	Ineligible outcome	O	Outcomes reflected substance use/co-use patterns rather than SUD/addiction diagnoses or overdose events.

Notes. PEO, Population–Exposure–Outcome; SUD, substance use disorder. Exclusions were based on full-text assessment against the prespecified outcome definitions and the requirement for an extractable exposure–outcome association estimate.

**Table 7:** Characteristics of included studies (n = 9).

Study	Country/Setting (Data Source)	Design	Population Frame (P)	Analytic Sample Size (N)	Early Adversity Measure (E)	Eligible Outcome(s) (O)
Gao et al. (2010)	USA; psychiatric clinical sample (STEP-BD rapid-cycling bipolar disorder)	Analytical cross-sectional	Psychiatric treatment sample	568	Childhood abuse/early trauma history (physical/sexual/verbal; single-item history measures)	SUD/addiction outcomes (lifetime and/or recent; substance-specific where reported)
Lake et al. (2015)	Canada; Vancouver cohorts (VIDUS + ACCESS)	Prospective cohort	Community cohort (PWID)	1697	Childhood Trauma Questionnaire (CTQ) domains/subscales	Non-fatal overdose
Stein et al. (2017)	USA; opioid use disorder treatment setting	Analytical cross-sectional	Psychiatric/SUD treatment sample (OUD)	457	ACE total score (0–10), continuous (per +1 ACE)	Non-fatal overdose history (lifetime)
Bryant et al. (2020)	USA; safety-net behavioural health/primary care setting	Analytical cross-sectional	Psychiatric/behavioural health clinical service sample	4378	ACE count (categorical) and/or ACE items (as reported)	Clinically recorded SUD diagnosis (any SUD and/or specific SUDs, as reported)
Moss et al. (2020)	USA; Add Health (national cohort)	Longitudinal cohort analysis (secondary data)	Community cohort followed into young adulthood	15,356 (baseline); analytic N NR	ACE burden (categorical) and developmental adversity indicators (as reported)	SUD/addiction outcomes derived from survey-based diagnostic/severity constructs (AUD/TUD/CUD, as reported)
McCabe et al. (2022)	USA; NESARC-III (national survey)	Analytical cross-sectional	Community adults (general population survey)	36,309	Childhood sexual abuse (CSA) typology/number of CSA types (as reported)	DSM-5 SUD (any and/or substance-specific SUDs, as reported)
Tschampl et al. (2022)	USA; treatment-seeking sample (predominantly Latinx)	Analytical cross-sectional	SUD treatment-seeking adults	149 (final model NR)	ACE total score (0–10), continuous (per +1 ACE)	Non-fatal overdose history (lifetime)
Broekhof et al. (2023)	Norway; Young-HUNT cohort with national registry linkage	Prospective cohort (registry follow-up)	Community cohort (baseline adolescence; adult outcome ascertainment)	8199	ACEs (any ACE vs. none and/or accumulation score, as reported)	Adult SUD diagnosis (registry-based; alcohol and/or illicit drug use disorder, as reported)
Asheh et al. (2023)	USA; outpatient addiction/dual-diagnosis clinic	Analytical cross-sectional (retrospective chart-based)	Outpatient addiction/dual-diagnosis care	115	ACE total score (continuous; per +1 ACE)	Non-fatal overdose history

Notes. ACE, adverse childhood experiences; AUD, alcohol use disorder; CSA, childhood sexual abuse; CTQ, Childhood Trauma Questionnaire; NESARC-III, National Epidemiologic Survey on Alcohol and Related Conditions–III; OUD, opioid use disorder; PWID, people who inject drugs; SUD, substance use disorder; TUD, tobacco use disorder; CUD, cannabis use disorder; NR, not reported; N, analytic sample size, in the extracted text available for this draft. “Baseline adolescence; adult outcome ascertainment” indicates eligibility under the prespecified rule that outcomes must be measured in adulthood even if cohort recruitment occurred earlier.

**Table 8:** Risk of bias (JBI analytical cross-sectional checklist; included cross-sectional studies, n = 7).

Study	Q1	Q2	Q3	Q4	Q5	Q6	Q7	Q8	Global RoB	Main Issue(s) Driving RoB
Gao et al. (2010)	Y	Y	U	Y	Y	Y	Y	Y	Low	Exposure history based on retrospective reporting; limited detail on measurement properties.
Stein et al. (2017)	Y	Y	Y	N	Y	Y	U	Y	High	Overdose outcome not ascertained using objective/standard criteria; outcome validity partly unclear.
Bryant et al. (2020)	Y	Y	Y	Y	Y	Y	Y	Y	Low	Clinical service sample may limit representativeness; exposure relies on retrospective ACE reporting.
Moss et al. (2020)	Y	Y	U	Y	Y	Y	Y	Y	Low	Retrospective ACE exposure reconstructed from survey items; potential misclassification cannot be excluded.
McCabe et al. (2022)	Y	Y	U	Y	Y	Y	Y	Y	Low	Retrospective CSA exposure; cross-sectional design limits temporal ordering for some contrasts.
Tschampl et al. (2022)	Y	Y	Y	N	Y	Y	U	Y	High	Overdose outcome measured by self-report without objective/standard criteria; outcome validity partly unclear.
Asheh et al. (2023)	Y	Y	Y	N	Y	Y	U	Y	High	Overdose outcome based on recorded history/self-report; objective/standard outcome criteria not explicit.

Notes. JBI, Joanna Briggs Institute; RoB, risk of bias; Y, yes; N, no; U, unclear; ACE, adverse childhood experiences; CSA, childhood sexual abuse. Item definitions: Q1 inclusion criteria clearly defined; Q2 study subjects and setting described; Q3 exposure measured in a valid and reliable way; Q4 objective, standard criteria used for outcome measurement; Q5 confounding factors identified; Q6 strategies to deal with confounding stated; Q7 outcomes measured in a valid and reliable way; Q8 appropriate statistical analysis used. Global RoB followed the prespecified rule reported in Methods.

**Table 9:** Risk of bias (JBI cohort checklist; included cohort studies, n = 2).

Study	Q1	Q2	Q3	Q4	Q5	Q6	Q7	Q8	Q9	Q10	Q11	Global RoB	Main Issue(s) Driving RoB
Lake et al. (2015)	Y	Y	Y	Y	Y	U	U	Y	U	U	Y	High	Several follow-up domains were unclear (completeness, handling of attrition); overdose outcome relied on self-report.
Broekhof et al. (2023)	Y	Y	Y	Y	Y	Y	Y	Y	Y	Y	Y	Low	Registry-based outcome ascertainment reduced misclassification; residual concern relates to self-reported ACE exposure at baseline.

Notes. Item definitions: Q1 groups similar/recruited from same population; Q2 exposure measured similarly for groups; Q3 exposure measured validly and reliably; Q4 confounders identified; Q5 confounding addressed; Q6 outcome-free at baseline; Q7 outcome measured validly and reliably; Q8 follow-up time sufficient; Q9 follow-up complete; Q10 incomplete follow-up addressed; Q11 appropriate statistical analysis used. Global RoB followed the prespecified rule reported in Methods.

**Table 10:** Key extracted adjusted associations for the primary outcome (SUD/addiction).

Study	Population Frame	Exposure Contrast Selected	Primary Outcome Operationalisation	Adjusted Association (OR/aOR, 95% CI)	Extraction Note
Gao et al. (2010) (N = 568)	Psychiatric treatment sample (rapid-cycling bipolar disorder)	Childhood physical abuse (yes vs. no)	Substance use disorder (lifetime)	OR 1.71 (1.067–2.735)	Cross-sectional clinical sample; effect used to represent disorder-level SUD association.
Bryant et al. (2020) (N = 4378)	Behavioural health clinical service sample	High ACE burden (≥4 vs. 0)	Any SUD diagnosis	aOR 2.83 (1.95–4.09)	ACE operationalisation supports a high vs. none contrast; covariate set varies by model as reported.
Moss et al. (2020) (N = NR; baseline 15,356)	Community cohort (Add Health; longitudinal)	ACE burden (4+ vs. 0)	Severe alcohol use disorder (AUD)	OR 2.92 (1.33–6.40)	Severity-based AUD outcome; retained as a disorder-level addiction endpoint for structured synthesis.
McCabe et al. (2022) (N = 36,309)	Community adults (NESARC-III)	≥2 CSA types vs. none (female model)	DSM-5 SUD (any)	aOR 2.10 (1.50–2.90)	Stratified reporting; this contrast was selected as the most interpretable dose-related CSA comparison with extractable precision.
Broekhof et al. (2023) (N = 8199)	Community cohort with registry linkage (baseline adolescence; adult outcomes)	Any ACE vs. none	Adult SUD diagnosis (registry-based)	OR 4.30 (2.50–7.30)	Registry outcome reduces outcome misclassification; exposure measured at baseline as reported.

Notes. ACE, adverse childhood experiences; CSA, childhood sexual abuse; SUD, substance use disorder; AUD, alcohol use disorder; OR/aOR, (adjusted) odds ratio; CI, confidence interval. Values >1 indicate higher odds of the SUD/addiction outcome in the exposed group (or higher exposure category), based on each study’s coding. “Adjusted” refers to multivariable models as reported by the original study; covariate sets were not uniform across studies. N in the Study column denotes the analytic sample size as reported by the original study; NR indicates not reported.

**Table 11:** Key extracted adjusted associations for the secondary outcome (non-fatal overdose).

Study	Population Frame	Exposure Contrast Selected	Secondary Outcome Operationalisation	Adjusted Association (OR/aOR, 95% CI)	Extraction Note
Lake et al. (2015) (N = 1697)	Community cohort (PWID)	CTQ physical abuse (domain-based contrast, as reported)	Non-fatal overdose	OR 1.36 (1.08–1.71)	Exposure defined via CTQ domain rather than ACE count; retained for structured synthesis, not pooled with per-point ACE effects.
Stein et al. (2017) (N = 457)	SUD treatment sample (OUD)	ACE score (per +1 ACE)	Lifetime non-fatal overdose	OR 1.10 (1.02–1.20)	Comparable per-point ACE scaling.
Tschampl et al. (2022) (N = 149; final model N NR)	SUD treatment-seeking adults	ACE score (per +1 ACE)	Lifetime non-fatal overdose	OR 1.27 (1.07–1.51)	Comparable per-point ACE scaling.
Asheh et al. (2023) (N = 115)	Outpatient addiction/dual-diagnosis care	ACE score (per +1 ACE)	Non-fatal overdose history	aOR 1.23 (1.00–1.50)	Comparable per-point ACE scaling; outcome captured as history as reported.

Notes. ACE, adverse childhood experiences; CTQ, Childhood Trauma Questionnaire; OUD, opioid use disorder; PWID, people who inject drugs; OR/aOR, (adjusted) odds ratio; CI, confidence interval. Values > 1 indicate higher odds of non-fatal overdose with higher adversity exposure, based on each study’s coding. “Adjusted” refers to multivariable models as reported by the original study. N in the Study column denotes the analytic sample size as reported by the original study; NR indicates not reported.

**Table 12:** Effect sizes included in the non-fatal overdose meta-analysis (ACE score per +1 point; k = 3).

Study	Exposure Coding	Adjusted OR	95% CI	log (OR)	SE (log OR)
Stein et al. (2017)	ACE total score (0–10), per +1	1.10	1.02–1.20	0.0953	0.0415
Tschampl et al. (2022)	ACE total score (0–10), per +1	1.27	1.07–1.51	0.2390	0.0879
Asheh et al. (2023)	ACE total score, per +1	1.23	1.00–1.50	0.2070	0.1034

Notes. ACE, adverse childhood experiences; OR, odds ratio; CI, confidence interval; SE, standard error. log (OR) and SE (log OR) were derived from the 95% CI on the log scale for pooling.

**Table 13:** Random-effects meta-analysis for non-fatal overdose (ACE score per +1 point; k = 3).

Study	Adjusted OR (95% CI)	Weight (Random Effects)
Stein et al. (2017)	1.10 (1.02–1.20)	58.7%
Tschampl et al. (2022)	1.27 (1.07–1.51)	23.3%
Asheh et al. (2023)	1.23 (1.00–1.50)	18.0%
Pooled (random effects)	1.16 (1.06–1.28)	—

Notes. Random effects model: DerSimonian–Laird. Heterogeneity: Q = 2.79, I^2^ = 28.4%, τ^2^ = 0.00225. Hartung–Knapp sensitivity analysis: pooled OR 1.16 (0.95–1.41). With k = 3, funnel-plot asymmetry testing was not undertaken.

**Table 14:** ROB-ME assessment for the overdose meta-analysis (synthesis-level judgement; k = 3).

Synthesis	Planned Assessment	Quantitative Indicators Available?	Key Considerations (Missing Evidence)	ROB-ME Judgement
Overdose (ACE per +1 point)	ROB-ME (Page et al., 2023)	No (k < 10)	English-language restriction; substantial proportion of non-accessible full texts during screening; sparse evidence base (k = 3) limits detection of dissemination patterns; multi-database searching with no date limits partially mitigates under-ascertainment	Some concerns

Notes. ROB-ME, Risk of Bias due to Missing Evidence; k, number of studies contributing to the synthesis; ACE, adverse childhood experiences. “Some concerns” reflects that missing evidence bias cannot be excluded and cannot be quantified, given k = 3.

**Table 15:** Summary of findings (GRADE): non-fatal overdose outcome, per 1-point increase in ACE score.

Outcome	Studies (Design)	Participants *	Relative Association (Random Effects)	Certainty (GRADE)	Main Reasons for Rating Decisions
History/lifetime non-fatal overdose	3 (observational)	721	OR 1.16 (95% CI 1.06–1.28)	Low	Rated down for study limitations (risk of bias in contributing studies); rated down for imprecision (k = 3; Hartung–Knapp interval included the null) (Hartung & Knapp, 2001)

Notes. ACE, adverse childhood experiences; OR, odds ratio; CI, confidence interval. * Participant total reflects the sum of analytic samples reported for Stein et al. (2017), Tschampl et al. (2022), and Asheh et al. (2023); model-specific Ns may differ slightly across adjusted analyses. The Hartung–Knapp sensitivity analysis yielded OR 1.16 (95% CI 0.95–1.41), supporting the imprecision judgement (Hartung & Knapp, 2001).

**Table 16:** Sensitivity analyses for the non-fatal overdose meta-analysis (ACE score per +1 point; k = 3).

Analysis	Studies Included	Pooled OR	95% CI	Heterogeneity (I^2^)	Interpretation for Robustness
Random effects (DerSimonian–Laird)	Stein; Tschampl; Asheh	1.16	1.06–1.28	28.4%	Baseline pooled estimate (Section 3.5).
Random effects (Hartung–Knapp)	Stein; Tschampl; Asheh	1.16	0.95–1.41	—	Same point estimate; wider interval expected with sparse evidence (Hartung & Knapp, 2001; IntHout et al., 2014).
Leave-one-out (exclude Stein)	Tschampl; Asheh	1.25	1.10–1.43	0.0%	Pooled effect remains > 1; magnitude increases when the lowest-effect study is removed.
Leave-one-out (exclude Tschampl)	Stein; Asheh	1.12	1.04–1.21	0.5%	Pooled effect remains > 1; estimate shifts modestly towards the null.
Leave-one-out (exclude Asheh)	Stein; Tschampl	1.16	1.01–1.32	54.2%	Point estimate close to baseline; heterogeneity increases with k = 2.

Notes. ACE, adverse childhood experiences; OR, odds ratio; CI, confidence interval; k, number of studies. Hartung–Knapp refers to the Hartung–Knapp–Sidik–Jonkman variance adjustment used as a sensitivity approach for random-effects meta-analysis with sparse evidence (Hartung & Knapp, 2001; IntHout et al., 2014). I^2^ is reported where estimable; interpretation is unstable when k is very small.

**Table 17:** Direction of associations by outcome using prioritised study-level estimates.

Outcome Domain	Evidence Cluster	Studies (k)	Exposure Scaling (as Synthesised)	Direction	Range of Selected OR/aOR (95% CI)	Quantitative Synthesis Performed?
Overdose	ACE per +1 point	3	ACE total score, per +1	OR > 1 in all studies	1.10–1.27	Yes (random effects pooled OR 1.16)
Overdose	Trauma domain (CTQ)	1	CTQ domain contrast (as reported)	OR > 1	1.36	No (not commensurate with ACE per-point scaling)
SUD/addiction	Disorder-level outcomes (heterogeneous definitions)	5	Mixed contrasts (e.g., any ACE vs. none; ≥4 vs. 0; CSA types; abuse subtype)	OR > 1 in all selected estimates	1.71–4.30	No (incompatible exposure/outcome scaling across studies)

Notes. ACE, adverse childhood experiences; CTQ, Childhood Trauma Questionnaire; CSA, childhood sexual abuse; SUD, substance use disorder; OR/aOR, (adjusted) odds ratio; CI, confidence interval; k, number of studies contributing to the cluster. Ranges reflect the prioritised estimates selected for synthesis (Table 10 and Table 11) and are not intended to imply direct comparability across the SUD/addiction studies, given differences in exposure coding, outcome definitions, and adjustment sets.

**Table 18:** Proposed minimal reporting core to support commensurable evidence synthesis in this research area.

Domain	Minimum Items to Report	Why It Matters for Synthesis
Population/setting	Recruitment frame; clinical vs. community setting; SUD treatment context; country/health system; sample size; age/sex distribution	Supports transportability assessment and subgrouping by setting (clinical vs. community).
Exposure definition (adversity)	Instrument (ACE, CTQ, other); recall window; coding (continuous, categories, thresholds); timing (before 18); handling of missing items	Enables harmonisation of exposure contrast (e.g., per-point ACE, domain-specific trauma) and reduces construct drift.
Outcome definition (SUD/addiction)	Diagnostic framework (DSM/ICD); ascertainment (clinical diagnosis, administrative codes, validated scale, self-report); substance class; timeframe	Prevents mixing non-equivalent outcomes (diagnosis vs. symptoms vs. use) and improves comparability across studies.
Outcome definition (overdose)	Fatal vs. non-fatal; ascertainment source (self-report, clinical records, registry); timeframe (lifetime vs. past-year); substance involved if available	Essential to avoid pooling fatal and non-fatal outcomes and to interpret severity along the continuum.
Design and temporality	Study design; whether adversity assessment preceded outcome; follow-up period (if longitudinal)	Clarifies causal interpretability and supports sensitivity analyses by design type.
Confounding strategy	A priori confounder set; covariate definitions; justification (e.g., DAG); whether mediators were adjusted	Improves interpretability of adjusted estimates and reduces bias from over/under-adjustment.
Effect estimates	Report both unadjusted and adjusted estimates when feasible; specify contrast; scale (OR/RR/HR); per-unit scaling; standard errors/CI	Facilitates pooling and conversion across metrics and enables consistent weighting.
Reproducibility	Protocol/preregistration; full search strings; analytic code; data availability statement	Enables verification, updating, and transparent synthesis updates.

## Data Availability

The original contributions presented in this study are included in the article and Supplementary Materials. Further inquiries can be directed to the corresponding authors.

## Supplementary Materials

The following supporting information can be downloaded at: https://www.mdpi.com/article/10.3390/bs16040589/s1, File S1: Data extraction template; File S2: Extracted data and meta-analysis dataset; File S3: Analytic code and reproducibility notes (provided in TXT, DOCX, and XLSX formats); Table S1: Risk-of-bias decision log.

## Abbreviations

The following abbreviations are used in this manuscript:

ACE	Adverse childhood experiences
aOR	Adjusted odds ratio
AUD	Alcohol use disorder
CI	Confidence Interval
CSA	Childhood sexual abuse
CTQ	Childhood Trauma Questionnaire
CUD	Cannabis use disorder
DeCS	Health Sciences Descriptors
DSM-5	Diagnostic and Statistical Manual of Mental Disorders, 5th Edition
GRADE	Grading of Recommendations Assessment, Development and Evaluation
JBI	Joanna Briggs Institute
MeSH	Medical Subject Headings
MOOSE	Meta-analysis Of Observational Studies in Epidemiology
OR	Odds ratio
OUD	Opioid use disorder
PEO	Population, Exposure, Outcome
PRISMA	Preferred Reporting Items for Systematic Reviews and Meta-Analyses
PWID	People who inject drugs
RoB	Risk of bias
ROB-ME	Risk of Bias due to Missing Evidence
SMI	Severe mental illness
SUD	Substance use disorder
TUD	Tobacco use disorder
