# Supplementary material for: Early Adverse Experiences and the Likelihood of Substance Use Disorders and Non-Fatal Overdose in Clinical and Community Settings: A Systematic Review and Meta-Analysis
Source: Behav Sci (Basel). 2026 Apr 15;16(4):589. doi: 10.3390/bs16040589 (PMC13113328; doi:10.3390/bs16040589)
Supplement: Supplementary file 1 [file behavsci-16-00589-s001.zip › Supplementary_File_S3_Analytic_Code_and_Reproducibility.pdf]

Supplementary File S3. Analytic code and reproducibility notes (overdose meta-analysis)

This file provides a human-readable version of the analytic code used to reproduce the random-effects meta-analysis for the overdose outcome (ACE score per +1 point) reported in the manuscript (Tables 12-13 and Figure 2). Because some submission systems cannot open .py files, the code is included here as plain text.

Overview

Outcome: overdose history / lifetime overdose (secondary outcome).

Effect measure: adjusted odds ratio (OR) per 1-point increase in ACE score.

Model: random-effects (DerSimonian-Laird) with a Wald-type 95% CI; Hartung-Knapp sensitivity CI.

Input data

Input dataset: overdose_meta_analysis_data (provided separately as .xlsx/.csv). Expected columns include: Study, log (OR), SE (log OR).

Column	Definition
Study	Study label as cited in the manuscript.
Adjusted OR	Adjusted odds ratio as reported in the original study.
95% CI	95% confidence interval for the adjusted OR.
log (OR)	Natural logarithm of the adjusted OR.
SE (log OR)	Standard error of log (OR), derived from the 95% CI on the log scale.

Expected output (reference values)

Using the three studies in Table 12, the script reproduces the following summary statistics:

Statistic	Value

k (number of studies)	3
Pooled OR (DerSimonian-Laird)	1.16
95% CI (Wald)	1.06 to 1.28
Q	2.79
I^2 (%)	28.3%
tau^2	0.00224
Hartung-Knapp pooled OR (sensitivity)	1.16
Hartung-Knapp 95% CI	0.95 to 1.41

Note: With k=3, the Hartung-Knapp interval is wider and includes the null; this is reported in the manuscript as a sensitivity analysis.

Reproducibility notes

If you wish to execute the code locally, save the plain-text script as meta_analysis_overdose.py, place it in the same folder as the input dataset overdose_meta_analysis_data.csv (or export the .xlsx version to .csv), and run it using Python 3. Required packages: pandas; scipy (only needed for the Hartung-Knapp confidence interval).

Code listing (plain text)

The complete code listing is reproduced below. A runnable plain-text copy is also provided as meta_analysis_overdose.txt.

```
"""Reproducible computation for the overdose meta-analysis (ACE score per +1
point).


Input: overdose_meta_analysis_data.csv (exported from Table 12 in the
manuscript)

Output: Prints DerSimonian-Laird random-effects pooled OR (95% CI),

        heterogeneity statistics (Q, I^2, tau^2),

        and Hartung-Knapp (HK) random-effects CI.


Notes:

- This script is intended as a transparent reproducibility aid for the
manuscript.
```

```
- It assumes effect sizes are adjusted ORs with 95% CIs and derived log(OR) +
SE.

"""


import math

import pandas as pd


def dersimonian_laird(log_y, se):

    """DerSimonian-Laird random-effects meta-analysis on log scale."""

    w_fixed = [1/(s*s) for s in se]

    y_fixed = sum(w*y for w,y in zip(w_fixed, log_y)) / sum(w_fixed)

    Q = sum(w*(y - y_fixed)**2 for w,y in zip(w_fixed, log_y))

    df = len(log_y) - 1

    C = sum(w_fixed) - (sum(w_fixed_i**2 for w_fixed_i in w_fixed) /
sum(w_fixed))

    tau2 = max(0.0, (Q - df) / C) if C > 0 else 0.0

    w_re = [1/(s*s + tau2) for s in se]

    y_re = sum(w*y for w,y in zip(w_re, log_y)) / sum(w_re)

    se_re = math.sqrt(1 / sum(w_re))

    return y_re, se_re, Q, tau2


def i2(Q, k):

    df = k - 1

    if Q <= df or Q <= 0:

        return 0.0

    return max(0.0, (Q - df)/Q) * 100.0


def hartung_knapp_ci(y_re, se, log_y, tau2, alpha=0.05):

    """Hartung-Knapp (HK) adjustment for random-effects CI on log scale.

    Uses t_{k-1} with variance estimate based on residuals.
```

```
    """
    import scipy.stats as st

    k = len(log_y)
    w = [1/(s*s + tau2) for s in se]
    # Residual-based variance estimator
    # v = sum(w*(y - y_re)^2) / ((k-1)*sum(w))
    numerator = sum(w_i*(y - y_re)**2 for w_i,y in zip(w, log_y))
    v = numerator / ((k - 1) * sum(w))
    se_hk = math.sqrt(v)
    tcrit = st.t.ppf(1 - alpha/2, df=k-1)
    lo = y_re - tcrit*se_hk
    hi = y_re + tcrit*se_hk
    return lo, hi

def main():
    df = pd.read_csv("overdose_meta_analysis_data.csv")
    # Expected columns include: log (OR), SE (log OR)
    # Tolerate minor header variations
    log_col = next(c for c in df.columns if "log" in c.lower())
    se_col = next(c for c in df.columns if c.lower().startswith("se"))
    log_y = df[log_col].astype(float).tolist()
    se = df[se_col].astype(float).tolist()
    k = len(log_y)

    y_re, se_re, Q, tau2 = dersimonian_laird(log_y, se)
    I2 = i2(Q, k)

    # Wald-type (normal) CI
```

```
    lo = y_re - 1.96*se_re

    hi = y_re + 1.96*se_re



    print(f"k = {k}")

    print(f"DerSimonian-Laird random-effects pooled log(OR) = {y_re:.4f}")

    print(f"SE = {se_re:.4f}")

    print(f"Pooled OR = {math.exp(y_re):.2f} (95% CI {math.exp(lo):.2f}-
{math.exp(hi):.2f})")

    print(f"Q = {Q:.2f}")

    print(f"tau^2 = {tau2:.5f}")

    print(f"I^2 = {I2:.1f}%")



    # HK CI (requires scipy)

    try:

        hk_lo, hk_hi = hartung_knapp_ci(y_re, se, log_y, tau2)

        print(f"Hartung-Knapp pooled OR = {math.exp(y_re):.2f} (95% CI
{math.exp(hk_lo):.2f}-{math.exp(hk_hi):.2f})")

    except Exception as e:

        print("Hartung-Knapp CI not computed (scipy required):", e)



if __name__ == "__main__":

    main()
```
