# Supplementary material for: Early Adverse Experiences and the Likelihood of Substance Use Disorders and Non-Fatal Overdose in Clinical and Community Settings: A Systematic Review and Meta-Analysis
Source: Behav Sci (Basel). 2026 Apr 15;16(4):589. doi: 10.3390/bs16040589 (PMC13113328; doi:10.3390/bs16040589)
Supplement: Supplementary file 1 [file behavsci-16-00589-s001.zip › Supplementary_Table_S1_RoB_Decision_Log.pdf]

Supplementary Table S1. Risk-of-bias decision log (key non-Yes judgements and rationale).

Study	Global RoB	Non-Yes items (JBI checklist)	Rationale for non-Yes judgements (summary)
Gao et al. (2010)	Low	Q3=U	Exposure history based on retrospective reporting; limited detail on measurement properties.
Lake et al. (2015)	High	Q6=U, Q7=U, Q9=U, Q10=U	Several follow-up domains were unclear (completeness, handling of attrition); overdose outcome relied on self-report.
Stein et al. (2017)	High	Q4=N, Q7=U	Overdose outcome not ascertained using objective/standard criteria; outcome validity partly unclear.
Bryant et al. (2020)	Low	None (all items Y)	Clinical service sample may limit representativeness; exposure relies on retrospective ACE reporting.
Moss et al. (2020)	Low	Q3=U	Retrospective ACE exposure reconstructed from survey items; potential misclassification cannot be excluded.
McCabe et al. (2022)	Low	Q3=U	Retrospective CSA exposure; cross-sectional design limits temporal ordering for some contrasts.
Tschampl et al. (2022)	High	Q4=N, Q7=U	Overdose outcome measured by self-

			report without objective/standard criteria; outcome validity partly unclear.
Broekhof et al. (2023)	Low	None (all items Y)	Registry-based outcome ascertainment reduced misclassification; residual concern relates to self-reported ACE exposure at baseline.
Asheh et al. (2023)	High	Q4=N, Q7=U	Overdose outcome based on recorded history/self-report; objective/standard outcome criteria not explicit.

Notes. Q numbering follows the JBI analytical cross-sectional checklist (Table 8) and JBI cohort checklist (Table 9). Y, yes; N, no; U, unclear; RoB, risk of bias.
